# Fourteen years of continuous soil moisture records from plant and biocrust-dominated microsites

**DOI:** 10.1038/s41597-021-01111-6

**Published:** 2022-01-20

**Authors:** Joaquín Moreno, Sergio Asensio, Miguel Berdugo, Beatriz Gozalo, Victoria Ochoa, David S. Pescador, Blas M. Benito, Fernando T. Maestre

**Affiliations:** 1 Instituto Multidisciplinar para el Estudio del Medio “Ramon Margalef”, Universidad de Alicante, Edificio Nuevos Institutos, Carretera de San Vicente del Raspeig s/n, 03690 San Vicente del Raspeig, Spain; 2 Institut Department of Environmental Systems Science, ETH Zürich, Universitätstrasse 16, 8092 Zurich, Switzerland; 3 Departamento de Farmacología, Farmacognosia y Botánica, Facultad de Farmacia, Universidad Complutense de Madrid, Madrid, Spain; 4 Departamento de Biología y Geología, Física y Química Inorgánica, Escuela Superior de Ciencias Experimentales y Tecnológicas, Universidad Rey Juan Carlos, Calle Tulipán s/n, 28933 Móstoles, Madrid, Spain; 5 Departamento de Ecología, Universidad de Alicante, Carretera de San Vicente del Raspeig s/n, 03690 San Vicente del Raspeig, Alicante, Spain

**Keywords:** Hydrology, Ecosystem ecology

## Abstract

Drylands cover ~41% of the terrestrial surface. In these water-limited ecosystems, soil moisture contributes to multiple hydrological processes and is a crucial determinant of the activity and performance of above- and belowground organisms and of the ecosystem processes that rely on them. Thus, an accurate characterisation of the temporal dynamics of soil moisture is critical to improve our understanding of how dryland ecosystems function and are responding to ongoing climate change. Furthermore, it may help improve climatic forecasts and drought monitoring. Here we present the MOISCRUST dataset, a long-term (2006–2020) soil moisture dataset at a sub-daily resolution from five different microsites (vascular plants and biocrusts) in a Mediterranean semiarid dryland located in Central Spain. MOISCRUST is a unique dataset for improving our understanding on how both vascular plants and biocrusts determine soil water dynamics in drylands, and thus to better assess their hydrological impacts and responses to ongoing climate change.

## Abstract (summary)

**Table:** 

Measurement(s)	soil moisture
Technology Type(s)	soil moisture sensors
Factor Type(s)	temporal interval
Sample Characteristic - Environment	semi-arid grassland
Sample Characteristic - Location	Central Spain

Machine-accessible metadata file describing the reported data: 10.6084/m9.figshare.16951723

## Background & Summary

Drylands, which comprise all areas with an aridity index (precipitation divided by potential evapotranspiration) lower than 0.65, collectively form the largest set of biomes on Earth^1^. In these water-limited ecosystems^2,3^, soil moisture is a key determinant of their structure and functioning^4–6^ as it largely drives the activity of vascular plants and soil organisms^7^, and impacts multiple hydrological processes, such as runoff, evaporation and transpiration from vegetation^8^, and biogeochemical cycles^9^. As such, soil moisture largely affects essential ecosystem services provided by these ecosystems, such as soil fertility and biomass/food production, which directly sustain the livelihoods of more than 1 billion people worldwide^10^.

Soil moisture is characterized by complex dynamics across a wide range of spatio-temporal scales^11^. Thus, an accurate characterization of the spatio-temporal dynamics of soil moisture can be particularly helpful for assimilation models, weather and flood forecasting, surface and subsurface hydrology studies and drought monitoring at local and regional scales^8,9,11–13^. Moreover, it may widen our understanding on feedback mechanisms between different meteorological and hydrological components and their interaction with ongoing climate change^14^. Climate models forecast average (median) warming values ranging from 3.2 °C to 3.7 °C for drylands by the late XXI century^15^, which together with associated changes in rainfall patterns, may decrease soil moisture across drylands worldwide^16,17^. These projections are not, however, free from uncertainties^18^. Continuous and long-term (>10 yrs) observations of soil moisture are particularly valuable for calibrating remote sensing products^19^ and parameterizing hydrological/ecosystem models^12,13,20^. These observations can be particularly useful to reduce the uncertainty of forecasts of long-term changes in soil moisture and other hydrological and vegetation attributes due to climate change^20^. However, such soil moisture series are only available for a limited set of ecosystems and geographical areas^19,21^, and are particularly scarce in drylands.

In drylands, vegetation is typically organised in a two-phase mosaic composed by plant-covered patches interspersed in a matrix of open areas without perennial vascular plants^22–24^. Vegetated and open areas have contrasted water dynamics, with infiltration rates that are typically higher beneath plant patches which also have lower water losses via run-off and evaporation^25–28^. Open areas are, however, not devoid of life as they are commonly covered by biocrusts, communities dominated by mosses, lichens, fungi, and cyanobacteria living in the soil surface across drylands worldwide^29^. Both vascular plants and biocrusts are key modulators of the water cycle in drylands, as they affect processes that, such as infiltration, runoff and evapotranspiration^30^, ultimately determine soil moisture contents. Despite the hydrological importance of both vascular plants and biocrusts, no dataset characterizing long-term (>10 yr) temporal variations in soil moisture across plant- and biocrust-dominated areas (microsites) is currently available.

Here we introduce the MOISCRUST dataset, a 14-yr continuous dataset of surface soil moisture measurements from multiple microsites (vegetated and open areas with different degree of biocrust development) gathered from the Aranjuez Experimental Station, a semi-arid grassland in Central Spain where multiple studies on the ecology of biocrusts have been carried out^27,31–36^.

## Methods

### Study site

**Fig. 1:**
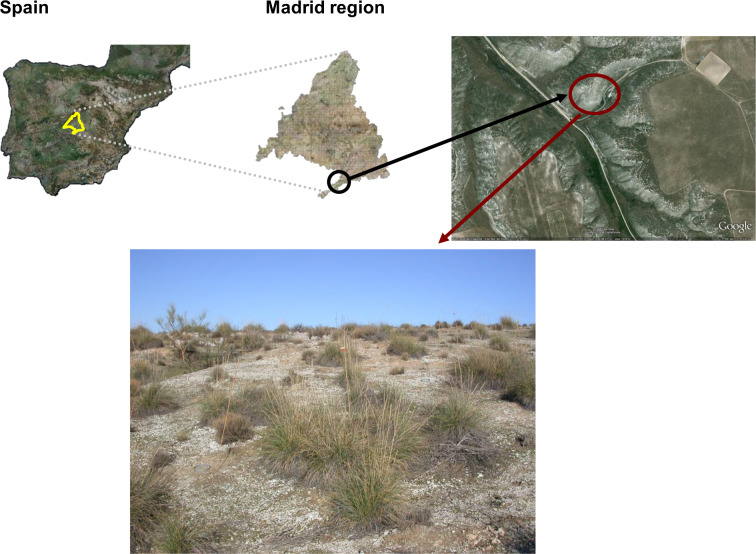
Location (upper panels) and partial view (lower panel) of the study area in central Spain, where patches of *Stipa tenacissima* and *Retama sphaerocarpa* are surrounded by a well-developed biocrust (white patches dominating the space between plant individuals) dominated by species such as *Diploschistes diacapsis*, *Fulgensia subbracteata* and *Psora decipiens*. From Berdugo *et al*.^7^.

The Aranjuez Experimental Station is located at the centre of the Iberian Peninsula (40° 02′ N–3° 32′ W; 590 m a.s.l., Fig. 1). The climate is Mediterranean semiarid, with average annual temperature and rainfall of 15 °C and 349 mm, respectively. Soils are classified as Gypsiric Leptosols^37^, with pH, organic carbon, and total nitrogen content values ranging between 7.2 and 7.7 mg/g, 9 and 32 mg/g, and 0.8 and 4 mg/g soil, respectively, depending on the microsite (open areas, vegetation, and biocrusts) considered^31^. Soils have a silty loam texture, showing c. 64.5%, 63.7–64.1% and 61.3–63.7% of sand, c. 28.4%, 28.4–29.2% and 30.0–32.4% of silt and c. 7.1%, 6.7–7.9% and 6.3% of clay for open and biocrust-dominated areas, respectively. The vegetation is dominated by *Stipa tenacissima* L. (18% of total cover), *Retama sphaerocarpa* (L.) Boiss, and *Helianthemun squamatum* Pers. (6% of total cover for both shrubs)^31^. The open areas between vascular plant patches are covered with a well‐developed biocrust community that covers ~34% of the soil surface, and is dominated by lichens such as *Diploschistes diacapsis* (Ach.) Lumbsch, *Squamarina lentigera* (Weber) Poelt, *Fulgensia subbracteata* (Nyl.) Poelt, *Toninia sedifolia* (Scop.) Timdal, and *Psora decipiens* (Hedw.) Hoffm^33^.

### Workflow

The reproducible workflow is available in the Supplementary Material as an interactive Rstudio notebook in the file *moiscrust.Rmd*. It is packaged with *renv* to facilitate reproducibility. That means that the R package versions originally used to run the notebook are already installed in the “renv” folder of the repository. This workflow contains the following steps: (i) Data loading and preparation, (ii) imputation of missing data, (iii) incorporating weather data at daily resolution, and (iv) preparing dataset formats (see Supplementary Material for more details).

## Data acquisition

**Fig. 2:**
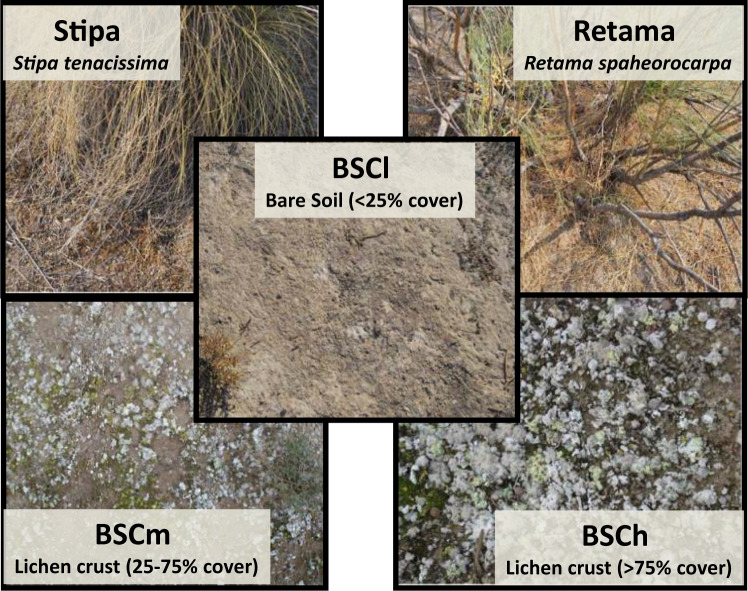
Photographs of the different microsites used in the study. Stipa = *Stipa tenacissima*; Retama = *Retama sphaerocarpa*; BSCl = open areas devoid of perennial vegetation with very low (<5%) cover of biocrust-forming lichens; BSCm = open areas with medium (25%–75%) cover of biocrust-forming lichens; BSCh = open areas with high (>75%) cover of biocrust-forming lichens. From Berdugo *et al*.^7^.

Soil moisture was measured in the five most common microsites at the study site (Fig. 2): *Stipa* tussocks (Stipa), *Retama* shrubs (Retama), and open areas devoid of perennial vegetation with very low (<5%, BSCl), medium (25%-75%, BSCm) and high (>75%, BSCh) cover of biocrust-forming lichens. *Stipa* microsites were placed at the north-face of *Stipa* tussocks, within 10 cm of their base, and are characterized by shaded conditions and a biocrust community dominated by mosses (mainly *Pleurochaete squarrosa* and *Tortula revolvens*). Retama microsites occur beneath the canopy of *R. sphaerocarpa* shrubs, and are characterized by moderate shade and litter accumulation. All microsites were selected in flat areas to reduce water retention from runoff, as this could be a confounding factor in soil moisture measurements, and were separated at least 2 m from one another.

**Fig. 3:**
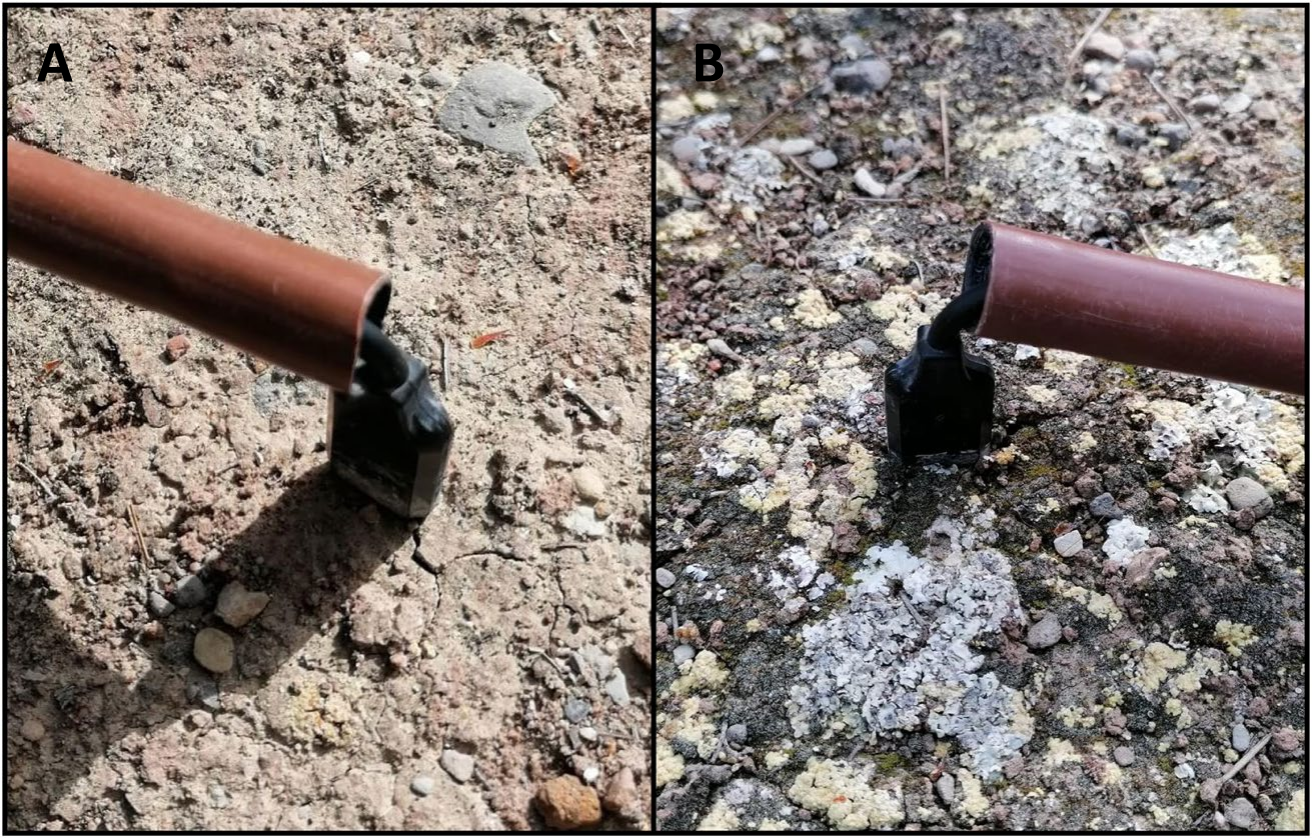
Pictures of the EC-5 moisture sensors used in open areas devoid of perennial vegetation with very low (<5%, **A**) and high (>75%, **B**) cover of biocrust-forming lichens.

We used soil moisture sensors (ECH2O EC-5, Decagon Devices Inc., Pullman, USA) to monitor soil moisture at sub-daily resolution. The sensors used provide estimates of volumetric water content (VWC) with an accuracy of ± 3%, and standard equations applied were used to sensor calibration in all microsites, as given their very similar texture values errors would be the same between microsites^38–44^. Such an approach has commonly been used in studies assessing soil moisture in drylands^38–42^, and works pretty well with the type of soils of our study site^43,44^. Three replicated sensors per microsite (total n = 15) were installed according to a stratified random design in November 2006 (Fig. 3). The sensors were introduced vertically in the soil^45^, so that the probe registered soil moisture from 0 to 5 cm depth. We did so for two main reasons: i) we were particularly interested in register the soil moisture in the topsoil (from 0 to 5 cm depth), which is the fraction of the soil profile particularly affected by plants and biocrusts (e.g^38,39,46–48^.), and ii) installing the sensors horizontally would have implied conducting substantial disturbance in a protected and very sensitive ecosystem (biocrusts are very sensitive to trampling and other disturbances^48–50^), and this was something we wanted to avoid at all costs. Doing so would have also affected other measurements we have been conducted in this experiment, such as soil respiration^46^. The study area also had a meteorological station (Onset, Pocasset, MA, USA) that collect daily temperature, precipitation and relative air humidity (error of ± 0.2 °C; ± 0.2 mm and ± 3.5% respectively) from 30^th^ March 2007 to 16^th^ December 2020. Besides, solar radiation (W/m²) was daily collected during this period using a Silicon Pyranometer (Onset S-LIB-M003).

Soil moisture has been recorded at the five different microsites described above since 17^th^ November 2006. Three replicated soil moisture sensors were placed at each microsite, recording measures of VWC (m^3^/m^3^) continuously (every 120 min from 17^th^ November 2006 to 31^th^ January 2017 and every 150 min from 1^st^ February 2017 to 16^th^ December 2020). Hence, the data presented in this Data Descriptor includes a spatio-temporal continuous soil moisture dataset from 2006 to 2020, and shows the effect of both vegetation and biocrusts (with different degree of cover) on soil moisture during this period.

### Filling data gaps

MOISCRUST contains a total of 697,695 records over the study period, obtained from a total of 15 soil moisture sensors, of which 380,583 are either missing or negative values (54.5% of the total records). These missing values are due to diverse causes, including damaged sensors, sensors that were removed for maintenance, exhausted batteries or malfunction caused by rabbits (*Oryctolagus cuniculus*), which gnaw the wires of the sensors (after we discovered rabbits do this we protected wires with a plastic hose). Besides, the MOISCRUST database has several negative values (anomalous values by imbalances in the standard equation) falling within the margin of error of the sensors. These anomalous values were set to NA. In these cases, when an anomalous data was observed, we checked whether the sensor continued to measure correctly by comparison with another trustworthy sensor. Later, equal observed measurements were included in the dataset, and anomalous measurements were discarded.

To fill the gaps in the MOISCRUST dataset, we first found, for a given entry *y* with missing data at time *t*, the sensor *x* with data for *t* that is in the same type of microsite (if possible), has the longest duration in common, and shows the highest correlation with the sensor to which *y* belongs. Then we estimated the missing value *y* with a linear model *y* ~ *x*. To find the best possible candidate sensor (*x*) to estimate the missing data (*y*), we correlated all pairs of sensors and computed a selection score based on the following equation:

$${S}_{x}= \% {vc}_{x,y}+({R}_{x,y}^{2}\cdot 100)+\left\{100,\;{\rm{if}}\;{{microsite}}_{x}={{microsite}}_{y}\;{\rm{or}}\;{\rm{0,}}\;{\rm{otherwise}}\right\}$$

where *S*_*x*_ is the selection score of the candidate sensor *x*; *y* is the sensor with a missing value to be estimated; *x* is the sensor to be used as candidate predictor to estimate the missing value in *y*; %*vc*_*x*,*y*_ is the percent of common valid cases of the sensors *x* and *y*; *R*^2^_*x*,*y*_ is the Pearson’s R² of the common valid cases of the sensors *x* and *y*; and *microsite*_*x*_ and *microsite*_*y*_ are the respective microsites of the sensors *x* and *y*. During data imputation, the sensor with the higher selection score was used to estimate each missing value (see Supplementary Material for a detailed description and a worked example of this procedure).

To provide an indicator of imputation quality, the algorithm generates a new column named *interpolation quality*, where the observed values are marked with “1”, and the imputed values contain the correlation coefficient of the model used to estimate them (see Supplementary Material for details). After this process was completed, the number of missing values in the dataset was reduced to 133,881 records (19.2% of the total records). The imputation algorithm was implemented using the R software^51^ and the libraries ‘renv’^52^, ‘data.table’^53^, ‘janitor’^54^, ‘tidyverse’^55^, ‘kableExtra’^56^, ‘foreach’^57^, ‘doParallel’^58^, ‘readr’^59^, ‘writexl’^60^, ‘RSQLite’^61^, ‘zip’^62^, ‘knitr’^63^, and ‘DBI’^64^.

### Data structure

The raw and interpolated data sets of soil moisture provide records and estimations of soil moisture from 17^th^ November 2006 to 16^th^ December 2020 in four different formats: plain text (csv), SQLite, R (.Rdata), and Excel (.xlsx).

## Data Records

Raw and imputed data (in the “data” and “database” folders, respectively) are freely available from Figshare^65^. Data files come along with a metadata file with a brief description of the dataset. This dataset will be updated annually in Figshare to include data additions. In addition, the repository contains the “renv” folder to facilitate the reproducibility (see Methods and Supplementary Material). For a fully description of this database please see the Data Descriptor “Moreno, J., S. Asensio, M. Berdugo, B. Gozalo, V. Ochoa, D. S. Pescador, B. M. Benito & F. T. Maestre. 2022. Fourteen years of continuous soil moisture records from plant and biocrust-dominated microsites. *Scientific* *Data*, 10.1038/s41597-021-01111-6”.

## Technical Validation

**Fig. 4:**
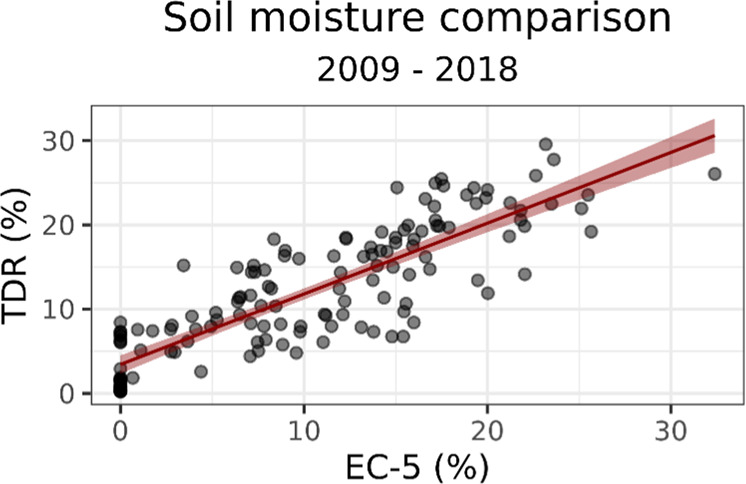
Relationship between soil moisture obtained by EC-5 sensors and Time Domain Reflectometry (TDR) measurements at the same date and microsite during 2009–2018.

Soil moisture measurements from the EC-5 sensors were validated using independent measurements obtained in the same date and microsites with the Time Domain Reflectometry technique (TDR^66^). These measurements were conducted at the same depth (0–5 cm) using TDR probes as described in Castillo-Monroy *et al*. . A total of 169 TDR measurements gathered between 17^th^ March 2009 to 25^th^ October 2018 and including the whole range of soil moisture values observed at the study area were used for this validation. The results obtained show a well-adjusted linear relationship between TDR and EC-5 measurements (adjusted R^2^ = 0.722, β = 0.839, 95% CI [0.753, 0.924], Fig. 4), which suggests that the sensors used properly measure soil moisture contents and their temporal variation at the study area.

## Usage Notes

Previous, short-term versions of the MOISCRUST dataset have been used to model annual variations in soil respiration rates across vegetation- and biocrust-dominated microsites, and to assess how vegetation, biocrusts and abiotic factors modulate wetting and drying events^7^. This dataset is particularly well suited for long-term studies focused on understanding spatio-temporal patterns of soil moisture in drylands^67^, and to analyse the effects of soil moisture–vegetation relationships (e.g. links between plant functional types and soil moisture^68^) and feedbacks on the dynamics of dryland ecosystems^69^. It also can be used to evaluate how both vascular plants and biocrusts determine soil water dynamics in drylands, to parameterize/tune up hydrological models aiming to study the hydrological behaviour of these ecosystems and to forecast their hydrological responses to ongoing climate change. Overall, the data provided by MOISCRUST contributes to advance our understanding of hydrologic processes in drylands and as such will be of interest to both researchers and managers working in these important ecosystems.

When using data from the MOISCRUST dataset please cite this publication. Both data and code are available under a Creative Commons Attribution 4.0 International Public License, whereby anyone may freely use data and adapt our dataset, as long as the original source is credited, the original license is linked, and any changes to our data are indicated in subsequent use.

## Supplementary information

Supplementary Material

## Data Availability

The code used for data imputation and dataset formatting is available in Figshare^65^.

## References

[1] Cherlet, M., *et al* (Eds.). *World Atlas of Desertification*. Luxembourg: Publication Office of the European Union (2018).

[2] Belnap J (2006). The potential roles of biological soil crusts in dryland hydrologic cycles. Hydrol. Process..

[3] Maestre FT (2011). Ecology and functional roles of biological soil crusts in semi-arid ecosystems of Spain. J. Arid Environ..

[4] Noy-Meir I (1973). Desert ecosystems: environment and producers. Annu. Rev. Ecol. Evol. Syst..

[5] Puigdefábregas J, Sole A, Gutierrez L, Del Barrio G, Boer M (1999). Scales and processes of water and sediment redistribution in drylands: results from the Rambla Honda field site in Southeast Spain. Earth-Sci. Rev..

[6] Puigdefábregas J (2005). The role of vegetation patterns in structuring runoff and sediment fluxes in drylands. Earth Surf. Process. Landf..

[7] Berdugo M, Soliveres S, Maestre FT (2014). Vascular plants and biocrusts modulate how abiotic factors affect wetting and drying events in drylands. Ecosystems.

[8] Meza FJ, Montes C, Bravo-Martínez F, Serrano-Ortiz P, Kowalski AS (2018). Soil water content effects on net ecosystem CO_2_ exchange and actual evapotranspiration in a Mediterranean semiarid savanna of Central Chile. Sci. Rep..

[9] Austin AT (2004). Water pulses and biogeochemical cycles in arid and semiarid ecosystems. Oecologia.

[10] Safirel, U & Adeel, Z. *Ecosystems and human well-being: current state and trends*, vol. 1. Washington, DC: Island Press (2005).

[11] Brocca L, Melone F, Moramarco T, Morbidelli R (2010). Spatial-temporal variability of soil moisture and its estimation across scales: Soil Moisture Spatiotemporal Variability. Water Resour. Res..

[12] Brocca L (2012). Assimilation of surface-and root-zone ASCAT soil moisture products into rainfall–runoff modeling. IEEE Trans. Geosci. Remote Sens..

[13] Parinussa R (2014). Global surface soil moisture from the Microwave Radiation Imager onboard the Fengyun-3B satellite. Int. J. Remote Sens..

[14] Cui Y (2019). A spatio-temporal continuous soil moisture dataset over the Tibet Plateau from 2002 to 2015. Sci. Data.

[15] Solomon, S. *et al*. (Eds.). *Climate Change 2007: The Physical Science Basis. Contribution of Working Group I to the Fourth Assessment Report of the Intergovernmental Panel in Climate Change*. Cambridge and New York: Cambridge University Press (2007).

[16] Soong JL, Phillips CL, Ledna C, Koven CD, Torn MS (2020). CMIP5 models predict rapid and deep soil warming over the 21st century. J. Geophys. Res. Biogeosci..

[17] Zhou S (2021). Soil moisture–atmosphere feedbacks mitigate declining water availability in drylands. Nat. Clim. Change.

[18] Lian, X. *et al*. Multifaceted characteristics of dryland aridity changes in a warming world. *Nat. Rev. Earth Environ*. **1**–**19** (2021).

[19] Naz BS, Kollet S, Franssen HJH, Montzka C, Kurtz W (2020). A 3 km spatially and temporally consistent European daily soil moisture reanalysis from 2000 to 2015. Sci. Data.

[20] Tietjen B (2010). Effects of climate change on the coupled dynamics of water and vegetation in drylands. Ecohydrology.

[21] Cui Y (2019). A spatio-temporal continuous soil moisture dataset over the Tibet Plateau from 2002 to 2015. Sci. Data.

[22] Tongway, D.J., Valentin, C., Seghieri, J. (Eds.). *Banded vegetation patterning in arid and semiarid environments: ecological processes and consequences for management*. Berlin: Springer (2001).

[23] Maestre FT, Cortina J (2002). Spatial patterns of surface soil properties and vegetation in a Mediterranean semi-arid steppe. Plant Soil.

[24] Maestre, F.T. *et al*. Biogeography of global drylands. *New Phytol*. (2021). 10.1111/nph.17395 33864276

[25] Bhark EW, Small EE (2003). Association between plant canopies and the spatial patterns of infiltration in shrubland and grassland of the Chihuahuan Desert, New Mexico. Ecosystems.

[26] Yepez EA (2005). Dynamics of transpiration and evaporation following a moisture pulse in semiarid grassland: a chamber-based isotope method for partitioning flux components. Agric. For. Meteorol..

[27] Eldridge DJ (2010). Interactive effects of three ecosystem engineers on infiltration in a semi-arid Mediterranean grassland. Ecosystems.

[28] Cerdà A (1997). The effect of patchy distribution of *Stipa tenacissima* L. on runoff and erosion. J. Arid Environ..

[29] Weber, B., Büdel, B. & Belnap, J. (Eds.). *Biological soil crusts: an organizing principle in drylands*. Cham: Springer (2016).

[30] Eldridge DJ (2020). The pervasive and multifaceted influence of biocrusts on water in the world’s drylands. Glob. Change Biol..

[31] Castillo-Monroy AP, Delgado-Baquerizo M, Maestre FT, Gallardo A (2010). Biological soil crusts modulate nitrogen availability in semi-arid ecosystems: Insights from a Mediterranean grassland. Plant Soil.

[32] Escolar C, Martínez I, Bowker MA, Maestre FT (2012). Warming reduces the growth and diversity of biological soil crusts in a semi-arid environment: implications for ecosystem structure and functioning. Philos. T. R. Soc. B..

[33] Maestre FT (2013). Changes in biocrust cover drive carbon cycle responses to climate change in drylands. Glob. Change Biol..

[34] Delgado‐Baquerizo M (2014). Direct and indirect impacts of climate change on microbial and biocrust communities alter the resistance of the N cycle in a semiarid grassland. J. Ecol..

[35] Delgado‐Baquerizo M (2015). Differences in thallus chemistry are related to species‐specific effects of biocrust‐forming lichens on soil nutrients and microbial communities. Funct. Ecol..

[36] Lafuente A, Berdugo M, Ladron de Guevara M, Gozalo B, Maestre FT (2018). Simulated climate change affects how biocrusts modulate water gains and desiccation dynamics after rainfall events. Ecohydrology.

[37] IUSS Working Group WRB. *World Reference Base for Soil Resources 2006*. World Soil Resources Reports No. 103. Rome, Italy: FAO (2006).

[38] Chamizo S, Cantón Y, Lázaro R, Domingo F (2013). The role of biological soil crusts in soil moisture dynamics in two semiarid ecosystems with contrasting soil textures. J. Hydrol..

[39] Chamizo S, Cantón Y, Rodríguez‐Caballero E, Domingo F (2016). Biocrusts positively affect the soil water balance in semiarid ecosystems. Ecohydrol..

[40] Dalton, M., Buss, P., Treijs, A. & Portmann, M. in *Irrigation Australia Limited Regional Conference* (Penrith Panthers, 2015).

[41] Francesca V, Osvaldo F, Stefano P, Paola RP (2010). Soil moisture measurements: Comparison of instrumentation performances. J. Irrig. Drain. Eng..

[42] Payero JO, Nafchi AM, Davis R, Khalilian A (2017). An Arduino-based wireless sensor network for soil moisture monitoring using Decagon EC-5 sensors. Open J. soil Sci..

[43] Payero JO, Qiao X, Khalilian A, Mirzakhani-Nafchi A, Davis R (2017). Evaluating the effect of soil texture on the response of three types of sensors used to monitor soil water status. JWARP.

[44] Sakaki, T., Limsuwat, A., Smits, K.M. & Illangasekare, T.H. Empirical two‐point α‐mixing model for calibrating the ECH2O EC‐5 soil moisture sensor in sands. *Water Resour. Res*. **44**(4) (2008).

[45] Sharma H, Shukla MK, Bosland PW, Steiner R (2017). Soil moisture sensor calibration, actual evapotranspiration, and crop coefficients for drip irrigated greenhouse chile peppers. Agric. Water Manag..

[46] Castillo-Monroy AP, Maestre FT, Rey A, Soliveres S, García-Palacios P (2011). Biological soil crust microsites are the main contributor to soil respiration in a semiarid ecosystem. Ecosyst..

[47] Steven B, Gallegos-Graves LV, Belnap J, Kuske CR (2013). Dryland soil microbial communities display spatial biogeographic patterns associated with soil depth and soil parent material. FEMS Microbiol. Ecol..

[48] Ding J, Eldridge DJ (2020). Biotic and abiotic effects on biocrust cover vary with microsite along an extensive aridity gradient. Plant Soil.

[49] Rodríguez-Caballero E (2018). Ecosystem services provided by biocrusts: from ecosystem functions to social values. J. Arid Envion..

[50] Zaady, E., Eldridge, D.J. & Bowker, M.A. in *Biological soil crusts: An organizing principle in drylands* (Springer, 2016).

[51] R Core Team. *R: A language and environment for statistical computing*. R Foundation for Statistical Computing, Vienna, Austria. https://www.R-project.org/ (2019).

[52] Ushey, K. *renv: Project Environments. R package version 0.13.2*. https://CRAN.R-project.org/package=renv (2021).

[53] Dowle, M. & Srinivasan, A. *data.table: Extension of ‘data.frame’. R package version 1.14.0*. https://CRAN.R-project.org/package=data.table (2021).

[54] Firke, S. *janitor: Simple Tools for Examining and Cleaning Dirty Data. R package version 2.1.0*. https://CRAN.R-project.org/package = janitor (2021).

[55] Wickham H (2019). Welcome to the tidyverse. J. Open Source Softw..

[56] Zhu, H. *kableExtra: Construct Complex Table with ‘kable’ and Pipe Syntax. R package version 1.3.4*. https://CRAN.R-project.org/package=kableExtra (2021).

[57] Microsoft & Weston, S. *foreach: Provides Foreach Looping Construct. R package version 1.5.1*. https://CRAN.R-project.org/package=foreach (2020).

[58] Microsoft Corporation & Weston, S. *doParallel: Foreach Parallel Adaptor for the ‘parallel’ Package. R package version 1.0.16*. https://CRAN.R-project.org/package=doParallel (2020).

[59] Wickham, H. & Hester, J. *readr: Read Rectangular Text Data. R package version 1.4.0*. https://CRAN.R-project.org/package=readr (2020).

[60] Ooms, J. *writexl: Export Data Frames to Excel ‘xlsx’ Format. R package version 1.4.0*. https://CRAN.R-project.org/package=writexl (2021).

[61] Müller, K., Wickham, H., James, D.A. & Falcon, S. *RSQLite: ‘SQLite’ Interface for R. R package version 2.2.7*. https://CRAN.R-project.org/package=RSQLite (2021).

[62] Csárdi, G., Podgórski, K. & Geldreich, R. *zip: Cross-Platform ‘zip’ Compression. R package version 2.1.1*. https://CRAN.R-project.org/package=zip (2020).

[63] Xie, Y. *knitr: A General-Purpose Package for Dynamic Report Generation in R. R package version 1.31*. https://CRAN.R-project.org/package=knitr (2021).

[64] R Special Interest Group on Databases (R-SIG-DB), Wickham, H. & Müller, K. *DBI: R Database Interface. R package version 1.1.1*. https://CRAN.R-project.org/package=DBI (2021).

[65] Moreno J (2021). figshare.

[66] Topp GC, Davis JL (1985). Measurement of soil water content using time-domain reflectometry (TDR): a field evaluation. Soil Sci. Soc. Am. J..

[67] Cantón Y, Solé-Benet A, Domingo F (2004). Temporal and spatial patterns of soil moisture in semiarid badlands of SE Spain. J. Hydrol..

[68] Breshears DD, Barnes FJ (1999). Interrelationships between plant functional types and soil moisture heterogeneity for semiarid landscapes within the grassland/forest continuum: a unified conceptual model. Landsc. Ecol..

[69] D’Odorico P, Caylor K, Okin GS, Scanlon TM (2007). On soil moisture–vegetation feedbacks and their possible effects on the dynamics of dryland ecosystems. J. Geophys. Res..

