# Supplementary material for: Fourteen years of continuous soil moisture records from plant and biocrust-dominated microsites
Source: Sci Data. 2022 Jan 20;9:14. doi: 10.1038/s41597-021-01111-6 (PMC8776732; doi:10.1038/s41597-021-01111-6)
Supplement: Supplementary file 1 — Supplementary Material [file 41597_2021_1111_MOESM1_ESM.pdf]

Fourteen years of continuous soil moisture records from plant and biocrust-dominated microsites

Joaquín Moreno[1,§] Sergio Asensio[1] Miguel Berdugo[1,2]
Beatriz Gozalo[1] Victoria Ochoa[1] David Sánchez-Pescador[3,4]
Blas M. Benito[1,†] Fernando T. Maestre[1,5]

[1] Instituto Multidisciplinar para el Estudio del Medio "Ramon Margalef", Universidad de Alicante, Edificio Nuevos Institutos, Carretera de San Vicente del Raspeig s/n, 03690 San Vicente del Raspeig, Spain.

[2] Institut Department of Environmental Systems Science, ETH Zürich. Universitätstrasse 16, 8092 Zurich, Switzerland.

[3] Departamento de Farmacología, Farmacognosia y Botánica, Facultad de Farmacia, Universidad Complutense de Madrid, Madrid, Spain.

[4] Departamento de Biología y Geología, Física y Química Inorgánica, Escuela Superior de Ciencias Experimentales y Tecnológicas, Universidad Rey Juan Carlos, Calle Tulipán s/n, 28933 Móstoles, Madrid, Spain.

[5] Departamento de Ecología, Universidad de Alicante, Carretera de San Vicente del Raspeig s/n, 03690 San Vicente del Raspeig, Alicante, Spain.

[§] Corresponding author: Joaquín Moreno <joaquin.moreno@ua.es>

[†] Code author: Blas M. Benito <blasbenito@gmail.com>

Code and reproducible workflow created by Blas M. Benito

Contents

1 Summary 3

2 Reproducing this notebook 4

3 Data loading and preparation 4

3.1 Loading the required libraries . . . 4

3.2 Value to replace negative values with . . . 5

3.3 Loading and preparing the raw data . . . 5

3.4 Formatting dates and times . . . 5

3.5 Reordering columns and arranging into long format . . . 7

3.6 Replacing negative numbers with NA . . . 9

3.7 Visualization of the raw data . . . 9

3.8 Number of NA per sensor . . . 12

4 Imputation of missing data 13

4.1 Developing criteria to find candidates for gap filling . . . 13

4.2 Generating the x and y matrices to impute missing values . . . 18

4.3 Data imputation, step by step . . . 20

4.4 Applying the imputation algorithm to the complete dataset . . . 22

4.5 Visualizing the interpolated time series . . . 27

5 Incorporating weather data at daily resolution 32

6 Preparing database formats 34

6.1 Format description . . . 34

6.1.1 moiscrust . . . 34

6.1.2 moiscrust_weather . . . 36

6.2 Saving the data base in different formats . . . 37

1 Summary

The **MOISCRUST** database contains volumetric water content (VWC, m^3/m^3) records captured by sensors EC-5 (Decagon Devices Inc., Pullman, USA) every 120 minutes (17th November 2006 to 31th January 2017) and 150 minutes (1st February 2017 to 16th December 2020) from three replicates in five different microsites (*Stipa* tussocks, *Retama* shrubs, and areas with bare soil, and medium and high cover of biocrust-forming lichens) located in The Aranjuez Experimental Station (central Iberian Peninsula, $40^{o}02'$ N, $3^{o}32'$W; 590 m.a.s.l). During the long time-span these sensors have been working, there have been periods when data capture has not possible due to technical issues, and as consequence, 34.63% of the database records are missing entries. This reproducible notebook describes in detail the method used to impute missing data in the **MOISCRUST** database.

2 Reproducing this notebook

This reproducible workflow is available as an interactive Rstudio notebook in the file **moiscrust.Rmd** stored in this repository. It is packaged with renv to facilitate reproducibility. That means that the R package versions originally used to run the notebook are already installed in the renv folder of the repository. To run it in your computer, execute the code chunk below to prepare the session. You will need to replace eval = FALSE with eval = TRUE in the header of the code chunk.

```
install.packages("renv")
renv::restore()
```

3 Data loading and preparation

3.1 Loading the required libraries

The following R libraries are required to run this notebook.

```
library("data.table")
library("dplyr")
library("magrittr")
library("tidyr")
library("ggplot2")
library("kableExtra")
library("foreach")
library("doParallel")
library("readr")
library("writexl")
library("RSQLite")
library("zip")
library("knitr")
library("DBI")
```

3.2 Value to replace negative values with

Humidity sensors may drift over time, and yield negative values instead of zero. These negative values can be either be interpreted as malfunctions, or as zero humidity. The variable below is used to replace negative soil humidity values throughout this workflow. When set to NA, the variable replace.negatives.with represents sensor malfunctions. It should be set to 0 if negatives are interpreted as zero humidity.

```
replace.negatives.with <- NA
```

3.3 Loading and preparing the raw data

The raw data, stored in the file *moiscrust_raw.csv* was compiled by the members of Maestre Lab from the data provided by the soil moisture sensors.

```
#loading the raw moiscrust dataset
moiscrust <- data.table::fread(file = "data/moiscrust_raw.csv") %>%
  as.data.frame()
```

3.4 Formatting dates and times

The raw data contains two fields representing time, namely *date* (year, month, and day), and *time* (hour, minute, and seconds). Below we format these fields according to the POSIX standard, and add new fields adding criteria to subset the data by time:

- *date_time*: Date and time in POSIX format.
- *date_time_id*: Unique identificator for each *date_time*.
- *year*: Year number.
- *year_day*: Day of the year.
- *month*: Month number.
- *month_day*: Day of the month.
- *week*: Week of the year.
- *week_day*: Day of the week.

```r
#date to Year-month-day
moiscrust$date <- format(
  as.POSIXct(
    strptime(
      moiscrust$date,
      "%d/%m/%Y",
      tz = ""
      )
    ),
  format = "%Y-%m-%d"
  )

#time to Hour-Minute
moiscrust$time <- format(
  as.POSIXct(
    strptime(
      moiscrust$time,
      "%H:%M:%S",
      tz = ""
      )
    ),
  format = "%H:%M"
  )

#joining date and time
moiscrust$date_time <- as.POSIXct(
  paste(
    moiscrust$date,
    moiscrust$time
    ),
  format="%Y-%m-%d %H:%M"
  )

#unique id for each observation
moiscrust$date_time_id <- 1:nrow(moiscrust)
```

```
#creating year, month, and day related columns
moiscrust$year <- lubridate::year(moiscrust$date)
moiscrust$year_day <- lubridate::yday(moiscrust$date)
moiscrust$month <- lubridate::month(moiscrust$date)
moiscrust$month_day <- lubridate::mday(moiscrust$date)
moiscrust$week <- lubridate::week(moiscrust$date)
moiscrust$week_day <- lubridate::wday(moiscrust$date)
```

3.5 Reordering columns and arranging into long format

At this point, the *MOISCRUST* data has 15 columns representing soils moisture measures (five microsites per three replicates per microsite), and 10 columns representing *time*. The data is also structured in what is known as a "wide format". Below we reorder these columns to facilitate arranging the data into a "long format".

```
#names of the sensors
sensors <- c(
  "retama_1",
  "retama_2",
  "retama_3",
  "stipa_1",
  "stipa_2",
  "stipa_3",
  "bare_soil_1",
  "bare_soil_2",
  "bare_soil_3",
  "biocrust_medium_1",
  "biocrust_medium_2",
  "biocrust_medium_3",
  "biocrust_high_1",
  "biocrust_high_2",
  "biocrust_high_3"
)

#reordering columns of moiscrust to have time in the left side
moiscrust <- moiscrust[, c(
```

```r
  "date_time",
  "date_time_id",
  "date",
  "time",
  "year",
  "year_day",
  "month",
  "month_day",
  "week",
  "week_day",
  sensors
)]

#to long format
moiscrust_long <- tidyr::pivot_longer(
  moiscrust,
  cols = all_of(sensors),
  names_to = "sensor",
  values_to = "soil_moisture"
)
```

date_time	date_time_id	date	time	year	year_day	month	month_day	week	week_day	sensor	soil_moisture
2006-11-17 18:00:00	1	2006-11-17	18:00	2006	321	11	17	46	6	retama_1	0.197
2006-11-17 18:00:00	1	2006-11-17	18:00	2006	321	11	17	46	6	retama_2	NA
2006-11-17 18:00:00	1	2006-11-17	18:00	2006	321	11	17	46	6	retama_3	NA
2006-11-17 18:00:00	1	2006-11-17	18:00	2006	321	11	17	46	6	stipa_1	0.132
2006-11-17 18:00:00	1	2006-11-17	18:00	2006	321	11	17	46	6	stipa_2	NA
2006-11-17 18:00:00	1	2006-11-17	18:00	2006	321	11	17	46	6	stipa_3	NA
2006-11-17 18:00:00	1	2006-11-17	18:00	2006	321	11	17	46	6	bare_soil_1	0.232
2006-11-17 18:00:00	1	2006-11-17	18:00	2006	321	11	17	46	6	bare_soil_2	NA
2006-11-17 18:00:00	1	2006-11-17	18:00	2006	321	11	17	46	6	bare_soil_3	NA
2006-11-17 18:00:00	1	2006-11-17	18:00	2006	321	11	17	46	6	biocrust_medium_1	0.205
2006-11-17 18:00:00	1	2006-11-17	18:00	2006	321	11	17	46	6	biocrust_medium_2	NA
2006-11-17 18:00:00	1	2006-11-17	18:00	2006	321	11	17	46	6	biocrust_medium_3	NA
2006-11-17 18:00:00	1	2006-11-17	18:00	2006	321	11	17	46	6	biocrust_high_1	0.121
2006-11-17 18:00:00	1	2006-11-17	18:00	2006	321	11	17	46	6	biocrust_high_2	NA
2006-11-17 18:00:00	1	2006-11-17	18:00	2006	321	11	17	46	6	biocrust_high_3	NA
2006-11-17 20:00:00	2	2006-11-17	20:00	2006	321	11	17	46	6	retama_1	0.195
2006-11-17 20:00:00	2	2006-11-17	20:00	2006	321	11	17	46	6	retama_2	NA
2006-11-17 20:00:00	2	2006-11-17	20:00	2006	321	11	17	46	6	retama_3	NA
2006-11-17 20:00:00	2	2006-11-17	20:00	2006	321	11	17	46	6	stipa_1	0.131
2006-11-17 20:00:00	2	2006-11-17	20:00	2006	321	11	17	46	6	stipa_2	NA

3.6 Replacing negative numbers with NA

The MOISCRUST database has several negative values, which are outside of the actual range of soil moisture the sensors can measure. To fix this issue and dismiss these values, we replace every negative values with NA (using the variable replace.negatives.with described at the beginning of this document).

```
moiscrust_long <- dplyr::mutate(
  moiscrust_long,
  soil_moisture = ifelse(
    soil_moisture < 0,
    replace.negatives.with,
    soil_moisture
  )
)
```

3.7 Visualization of the raw data

Having the data in long format facilitates plotting and manipulation. The figure below shows the raw data, with missing data represented by the white color.

```
ggplot(moiscrust_long) +
  facet_wrap(
    "year",
    scales = "free_x",
    ncol = 2
    ) +
  aes(
    x = year_day,
    y = sensor,
    fill = soil_moisture
    ) +
  geom_tile() +
  coord_cartesian(expand = FALSE) +
  theme_bw() +
  scale_fill_viridis_c(
```

```
  direction = -1,
  na.value = "gray50",
  option = "B"
  ) +
theme(legend.position = "bottom") +
ylab("") +
xlab("Day of the year") +
ggtitle("MOISCRUST database (raw data)") +
labs(fill = expression("Volumetric water content (m³ water / m³ soil)")) +
theme(legend.key.width = unit(1, "cm"))
```

MOISCRUST database (raw data)

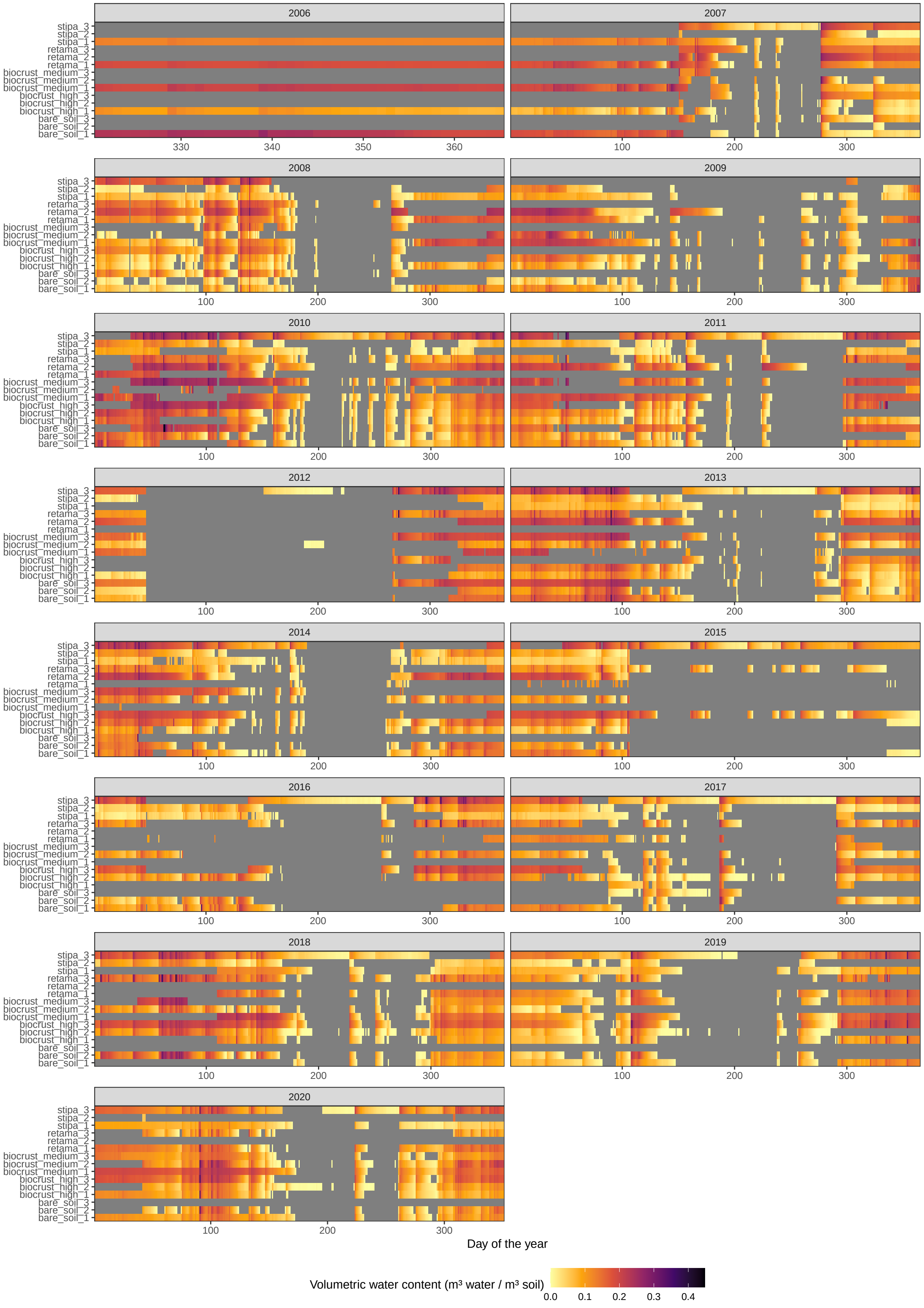

3.8 Number of NA per sensor

Due to technical constraints, there is a large number of missing data in the *MOISCRUST* dataset. To better understand the extent of such missing data, the code below counts the number of missing entries per sensor.

```
#counting NA values per sensor
moiscrust_NA <- moiscrust_long %>%
  group_by(sensor) %>%
  summarise(na_count = sum(is.na(soil_moisture))) %>%
  mutate(na_count_percent = round((na_count * 100) / nrow(moiscrust), 1))

#adding sensor microsite to the moiscrust_NA data frame
moiscrust_NA$microsite <- c(
  "bare_soil",
  "bare_soil",
  "bare_soil",
  "biocrust_high",
  "biocrust_high",
  "biocrust_high",
  "biocrust_medium",
  "biocrust_medium",
  "biocrust_medium",
  "retama",
  "retama",
  "retama",
  "stipa",
  "stipa",
  "stipa"
)

#reordering columns and arranging by na_count
moiscrust_NA <- moiscrust_NA[, c(
  "sensor",
  "microsite",
  "na_count",
  "na_count_percent"
```

```
)] %>%
dplyr::arrange(na_count) %>%
as.data.frame()
```

sensor	microsite	na_count	na_count_percent
stipa_3	stipa	13963	30.0
bare_soil_1	bare_soil	18442	39.6
biocrust_high_2	biocrust_high	18813	40.4
stipa_1	stipa	20840	44.8
biocrust_high_1	biocrust_high	22940	49.3
retama_3	retama	24007	51.6
biocrust_high_3	biocrust_high	24225	52.1
biocrust_medium_1	biocrust_medium	25797	55.5
bare_soil_2	bare_soil	27546	59.2
stipa_2	stipa	27698	59.5
biocrust_medium_2	biocrust_medium	29532	63.5
retama_1	retama	30111	64.7
biocrust_medium_3	biocrust_medium	30596	65.8
retama_2	retama	31366	67.4
bare_soil_3	bare_soil	34707	74.6

4 Imputation of missing data

The imputation we apply to fill gaps in *MOISCRUST* works by finding, for a given entry y with missing data at a time t, the sensor x with data for t that is in the same type of microsite (if possible), has the longest extent in common, and the highest correlation with the sensor to which y belongs. Then, y is estimated from the linear model $y\ x$.

4.1 Developing criteria to find candidates for gap filling

To generate the criteria to find the best possible candidate *x* to estimate the missing data *y*, we compute the common length and correlation between all pairs of sensors, and generate a column indicating whether they belong to the same microsite or not.

With the values stored in these columns we compute a *selection score* based on the following expression:

$$S_x = \%vc_{x,y} + (R^2_{x,y} * 100) + \begin{cases} 100, & \text{if } microsite_x == microsite_y \\ 0, & \text{otherwise} \end{cases}$$

Where:

- y is the sensor with a missing value to be estimated.
- x is the sensor to be used as candidate predictor to estimate the missing value in y.
- S_x is the selection score of the candidate sensor x, in the range [0, 300].
- $\%vc_{x,y}$ is the percent of common valid cases of the sensors x and y.
- $R^2_{x,y}$ is the Pearson's R^2 of the common valid cases of the sensors x and y.
- $microsite_x$ and $microsite_y$ are the respective microsites of the sensors x and y.

During data imputation, for each missing value, the sensor with the higher selection score is used to estimate it.

These criteria are stored in the data frame **sensors_pairs**. Along with the computation of the selection score, the code below also computes a linear model for each pair x y, and stores it in the object **sensors_pairs_models**. The identificators of these models are stored in the column *model_id* of the data frame **sensors_pairs**.

```
#combining sensors in pairs x-y
sensors_pairs <- combn(
  x = sensors,
  m = 2
) %>%
  t() %>%
  as.data.frame()

#adding combinations y-x so all pairs have both directions
#removing repeated pairs
#joining with moiscrust_NA to get sensor groups
#add column same_microsite to check if x and y are or not in the same sensor group
#add empty columns to store % of shared data, model's R squared, and model ID
sensors_pairs <- sensors_pairs %>%
```

```
rbind(
 data.frame(
  V1 = sensors_pairs$V2,
  V2 = sensors_pairs$V1
 )
) %>%
distinct(
 V1,
 V2,
 .keep_all = TRUE
) %>%
left_join(
 moiscrust_NA[, c("sensor", "microsite")],
 by = c("V1" = "sensor")
) %>%
left_join(
 moiscrust_NA[, c("sensor", "microsite")],
 by = c("V2" = "sensor")
) %>%
rename(
 y = V1,
 x = V2,
 y_microsite = microsite.x, #not a mistake
 x_microsite = microsite.y, #not a mistake
) %>%
mutate(
 same_microsite = ifelse(
  x_microsite == y_microsite,
  TRUE,
  FALSE
  ),
 sensors_shared_valid_percent = NA,
 sensors_r_squared = NA,
 model_id = row_number()
)
```

```
#list to store models
sensors_pairs_models <- list()

#looping through sensors pairs to:
#fit lm model y ~ x and save it in sensors_pairs_models
#
for(i in 1:nrow(sensors_pairs)){

  #names of the sensors y and x
  y_i <- sensors_pairs[i, "y"]
  x_i <- sensors_pairs[i, "x"]

  #data of the sensor pair
  sensor_pair_i <- moiscrust[, c(y_i, x_i)]

  #complete cases of the sensor pair
  sensor_pair_i <- sensor_pair_i[complete.cases(sensor_pair_i), ]

  #common cases
  sensors_pairs[i, "sensors_shared_valid_percent"] <-
    nrow(sensor_pair_i) / nrow(moiscrust) * 100

  #R squared of the sensor pair
  sensors_pairs[i, "sensors_r_squared"] <- cor(
    sensor_pair_i[, 1],
    sensor_pair_i[, 2]
    )

  #model formula y ~ x
  formula_i <- as.formula(paste(y_i, "~", x_i))

  #linear model
  sensors_pairs_models[[i]] <- lm(
    formula = formula_i,
    data = sensor_pair_i
  )
```

```
}

#selection score to find candidates during gap filling
#(sensors_r_squared * 100) +
#sensors_shared_valid_percent +
#same_microsite (TRUE = 100, FALSE = 0)
sensors_pairs <- mutate(
  sensors_pairs,
  selection_score =
    (sensors_r_squared * 100) +
    sensors_shared_valid_percent +
    ifelse(same_microsite == TRUE, 100, 0)
)

#removing objects we don't need
rm(
  sensor_pair_i,
  formula_i,
  i,
  x_i,
  y_i
)
```

The resulting data frame, named **sensors_pairs**, has columns with the names of the sensor *y* (the one with missing data to impute), the sensor *x* (the candidate to be used as predictor to estimate *y*), their respective microsites, a column indicating if they belong to the same microsite, the percent of shared valid data, the R squared of their shared data, a model ID linking each entry with a model in sensors_pairs_models, and a selection score used to select the best possible model to use in the imputation process.

y	x	y_microsite	x_microsite	same_microsite	sensors_shared_valid_percent	sensors_r_squared	model_id	selection_score
retama_1	retama_2	retama	retama	TRUE	19.49132	0.8875375	1	208.2451
retama_1	retama_3	retama	retama	TRUE	32.26840	0.9050852	2	222.7769
retama_1	stipa_1	retama	stipa	FALSE	49.39264	0.8768781	3	137.0805
retama_1	stipa_2	retama	stipa	FALSE	30.86449	0.8693809	4	117.8026
retama_1	stipa_3	retama	stipa	FALSE	32.64464	0.9077454	5	123.4192
retama_1	bare_soil_1	retama	bare_soil	FALSE	49.00995	0.8918511	6	138.1951
retama_1	bare_soil_2	retama	bare_soil	FALSE	40.65960	0.7716887	7	117.8285
retama_1	bare_soil_3	retama	bare_soil	FALSE	12.33204	0.8942298	8	101.7550
retama_1	biocrust_medium_1	retama	biocrust_medium	FALSE	45.66465	0.9131358	9	136.9782
retama_1	biocrust_medium_2	retama	biocrust_medium	FALSE	35.28691	0.7624165	10	111.5286
retama_1	biocrust_medium_3	retama	biocrust_medium	FALSE	21.96805	0.8633835	11	108.3064
retama_1	biocrust_high_1	retama	biocrust_high	FALSE	48.26823	0.8719324	12	135.4615
retama_1	biocrust_high_2	retama	biocrust_high	FALSE	43.13633	0.8749174	13	130.6281
retama_1	biocrust_high_3	retama	biocrust_high	FALSE	35.61585	0.9401687	14	129.6327
retama_2	retama_3	retama	retama	TRUE	35.53845	0.8520638	15	220.7448
retama_2	stipa_1	retama	stipa	FALSE	38.00658	0.7677740	16	114.7840
retama_2	stipa_2	retama	stipa	FALSE	38.66016	0.8345871	17	122.1189
retama_2	stipa_3	retama	stipa	FALSE	32.43824	0.8913270	18	121.5709
retama_2	bare_soil_1	retama	bare_soil	FALSE	44.80683	0.7984693	19	124.6538
retama_2	bare_soil_2	retama	bare_soil	FALSE	45.83235	0.7608700	20	121.9193

4.2 Generating the x and y matrices to impute missing values

During data imputation, two data frames are needed. The data frame **x** contains the data of every sensor for every available time, while the data frame **y**, which starts with empty values, is where the imputed values, their confidence intervals, selection criteria, and other quality-related columns are going to be stored.

```
#creating data frame of predictors
x <- moiscrust[, sensors]

#creating data frame to store model results
y <- matrix(
  data = NA,
  nrow = nrow(moiscrust),
  ncol = 12
  ) %>%
  as.data.frame()

#new colnames
colnames(y) <- c(
  "interpolated",
  "model_estimate",
  "model_ci_lower",
  "model_ci_upper",
```

```r
  "model_predictor",
  "same_microsite",
  "sensors_r_squared",
  "sensors_shared_valid_percent",
  "selection_score",
  "date_time_id",
  "sensor",
  "microsite"
  )

#transferring time id
y[, "date_time_id"] <- moiscrust[, "date_time_id"]
y[, "interpolated"] <- FALSE
```

The **x** data frame looks as follows:

retama_1	retama_2	retama_3	stipa_1	stipa_2	stipa_3	bare_soil_1	bare_soil_2	bare_soil_3	biocrust_medium_1	biocrust_medium_2	biocrust_medium_3	biocrust_high_1	biocrust_high_2	biocrust_high_3
0.197	NA	NA	0.132	NA	NA	0.232	NA	NA	0.205	NA	NA	0.121	NA	NA
0.195	NA	NA	0.131	NA	NA	0.233	NA	NA	0.203	NA	NA	0.117	NA	NA
0.194	NA	NA	0.131	NA	NA	0.234	NA	NA	0.203	NA	NA	0.117	NA	NA
0.194	NA	NA	0.131	NA	NA	0.234	NA	NA	0.203	NA	NA	0.116	NA	NA
0.194	NA	NA	0.130	NA	NA	0.234	NA	NA	0.203	NA	NA	0.116	NA	NA
0.194	NA	NA	0.130	NA	NA	0.234	NA	NA	0.202	NA	NA	0.116	NA	NA
0.194	NA	NA	0.130	NA	NA	0.234	NA	NA	0.202	NA	NA	0.115	NA	NA
0.194	NA	NA	0.130	NA	NA	0.234	NA	NA	0.202	NA	NA	0.115	NA	NA
0.194	NA	NA	0.130	NA	NA	0.234	NA	NA	0.202	NA	NA	0.115	NA	NA
0.194	NA	NA	0.130	NA	NA	0.234	NA	NA	0.202	NA	NA	0.116	NA	NA
0.195	NA	NA	0.131	NA	NA	0.234	NA	NA	0.202	NA	NA	0.116	NA	NA
0.195	NA	NA	0.131	NA	NA	0.233	NA	NA	0.201	NA	NA	0.115	NA	NA
0.194	NA	NA	0.130	NA	NA	0.233	NA	NA	0.200	NA	NA	0.114	NA	NA
0.193	NA	NA	0.129	NA	NA	0.233	NA	NA	0.199	NA	NA	0.112	NA	NA
0.192	NA	NA	0.129	NA	NA	0.234	NA	NA	0.199	NA	NA	0.111	NA	NA
0.192	NA	NA	0.128	NA	NA	0.234	NA	NA	0.199	NA	NA	0.111	NA	NA
0.191	NA	NA	0.128	NA	NA	0.234	NA	NA	0.198	NA	NA	0.110	NA	NA
0.190	NA	NA	0.127	NA	NA	0.234	NA	NA	0.198	NA	NA	0.109	NA	NA
0.189	NA	NA	0.127	NA	NA	0.234	NA	NA	0.198	NA	NA	0.109	NA	NA
0.188	NA	NA	0.127	NA	NA	0.234	NA	NA	0.198	NA	NA	0.109	NA	NA

This is the **y** data frame, that will be filled during the data imputation:

interpolated	model_estimate	model_ci_lower	model_ci_upper	model_predictor	same_microsite	sensors_r_squared	sensors_shared_valid_percent	selection_score	date_time_id	sensor	microsite
FALSE	NA	NA	NA	NA	NA	NA	NA	NA	1	NA	NA
FALSE	NA	NA	NA	NA	NA	NA	NA	NA	2	NA	NA
FALSE	NA	NA	NA	NA	NA	NA	NA	NA	3	NA	NA
FALSE	NA	NA	NA	NA	NA	NA	NA	NA	4	NA	NA
FALSE	NA	NA	NA	NA	NA	NA	NA	NA	5	NA	NA
FALSE	NA	NA	NA	NA	NA	NA	NA	NA	6	NA	NA
FALSE	NA	NA	NA	NA	NA	NA	NA	NA	7	NA	NA
FALSE	NA	NA	NA	NA	NA	NA	NA	NA	8	NA	NA
FALSE	NA	NA	NA	NA	NA	NA	NA	NA	9	NA	NA
FALSE	NA	NA	NA	NA	NA	NA	NA	NA	10	NA	NA
FALSE	NA	NA	NA	NA	NA	NA	NA	NA	11	NA	NA
FALSE	NA	NA	NA	NA	NA	NA	NA	NA	12	NA	NA
FALSE	NA	NA	NA	NA	NA	NA	NA	NA	13	NA	NA
FALSE	NA	NA	NA	NA	NA	NA	NA	NA	14	NA	NA
FALSE	NA	NA	NA	NA	NA	NA	NA	NA	15	NA	NA
FALSE	NA	NA	NA	NA	NA	NA	NA	NA	16	NA	NA
FALSE	NA	NA	NA	NA	NA	NA	NA	NA	17	NA	NA
FALSE	NA	NA	NA	NA	NA	NA	NA	NA	18	NA	NA
FALSE	NA	NA	NA	NA	NA	NA	NA	NA	19	NA	NA
FALSE	NA	NA	NA	NA	NA	NA	NA	NA	20	NA	NA

4.3 Data imputation, step by step

The steps to fill the gaps in the **MOISCRUST** database go as follows:

1. A given sensor name is selected: "retama_2"

```
sensor = "retama_2"
```

2. The sensors pairs from the table **sensors_pairs** where the selected sensor is *y* (the sensor which values are to be imputed) are selected.

```
sensors_pair <- sensors_pairs %>%
  dplyr::filter(y == sensor)
```

y	x	y_microsite	x_microsite	same_microsite	sensors_shared_valid_percent	sensors_r_squared	model_id	selection_score
retama_2	retama_3	retama	retama	TRUE	35.53845	0.8520638	15	220.7448
retama_2	stipa_1	retama	stipa	FALSE	38.00658	0.7677740	16	114.7840
retama_2	stipa_2	retama	stipa	FALSE	38.66016	0.8345871	17	122.1189
retama_2	stipa_3	retama	stipa	FALSE	32.43824	0.8913270	18	121.5709
retama_2	bare_soil_1	retama	bare_soil	FALSE	44.80683	0.7984693	19	124.6538
retama_2	bare_soil_2	retama	bare_soil	FALSE	45.83235	0.7608700	20	121.9193
retama_2	bare_soil_3	retama	bare_soil	FALSE	29.17034	0.8402989	21	113.2002
retama_2	biocrust_medium_1	retama	biocrust_medium	FALSE	31.35038	0.7975457	22	111.1049
retama_2	biocrust_medium_2	retama	biocrust_medium	FALSE	33.47666	0.7376920	23	107.2459
retama_2	biocrust_medium_3	retama	biocrust_medium	FALSE	27.62454	0.8515719	24	112.7817
retama_2	biocrust_high_1	retama	biocrust_high	FALSE	45.04762	0.7545435	25	120.5020
retama_2	biocrust_high_2	retama	biocrust_high	FALSE	45.04977	0.8237431	26	127.4241
retama_2	biocrust_high_3	retama	biocrust_high	FALSE	31.08808	0.8414304	27	115.2311
retama_2	retama_1	retama	retama	TRUE	19.49132	0.8875375	106	208.2451

3. The first row of the data frame **x** is selected.

```
x_row <- x[1, ]
```

retama_1	retama_2	retama_3	stipa_1	stipa_2	stipa_3	bare_soil_1	bare_soil_2	bare_soil_3	biocrust_medium_1	biocrust_medium_2	biocrust_medium_3	biocrust_high_1	biocrust_high_2	biocrust_high_3
0.197	NA	NA	0.132	NA	NA	0.232	NA	NA	0.205	NA	NA	0.121	NA	NA

3.1. If there is a valid value of soil moisture for the sensor "retama_2", the algorithm goes to the next row, until there is a row with a missing value.

4. If there is a missing value (*NA*), the potential candidate predictors are selected from the row by removing the data of other sensors with *NA*, and the data of the target sensor.

```
predictor_candidates <- as.vector(x[1, ])
predictor_candidates <- predictor_candidates[which(
    !is.na(predictor_candidates) &
```

```
    names(predictor_candidates) != sensor
  )]
```

retama_1	stipa_1	bare_soil_1	biocrust_medium_1	biocrust_high_1
0.197	0.132	0.232	0.205	0.121

5. From these predictors, the one with the highest selection score is selected from the data frame **sensors_pair** generated in the step **2.**.

```
best_predictor <- sensors_pair %>%
  dplyr::filter(x %in% names(predictor_candidates)) %>%
  dplyr::arrange(desc(selection_score)) %>%
  dplyr::slice(1)
```

y	x	y_microsite	x_microsite	same_microsite	sensors_shared_valid_percent	sensors_r_squared	model_id	selection_score
retama_2	retama_1	retama	retama	TRUE	19.49132	0.8875375	106	208.2451

6. The model to use, stored in the list **sensors_pairs_model**, is selected from *model_id* column of the **best_predictor** data frame , and used to predict a value for the empty cell.

```
predict(
  object = sensors_pairs_models[[best_predictor$model_id]],
  newdata = x_row,
  se.fit = TRUE,
  type = "response",
  interval = "confidence"
  )$fit
```

```
##      fit       lwr       upr
## 1 0.24973 0.2477106 0.2517494
```

After this step, if any of these values is negative, then they are replaced with `replace.negatives.with`. And if `replace.negatives.with` is `NA` and fit is negative, then all three outcomes of predict are replaced with `NA`.

7. The imputed value, its confidence intervals, and other values about the imputation quality available in **best_predictor** are transferred to the same row in the data frame **y**.

8. Once all the sensors and rows have been processed this way, the matrix **y** is joined with **moiscrust_long**, and its interpolated values are transferred to the *soil_moisture* column, along with other columns indicating the quality of the interpolation.

4.4 Applying the imputation algorithm to the complete dataset

The code below applies the algorithm explained above to every sensor and row with missing data. Sensors are processed in parallel to speed up the data imputation operation.

```
#setup for parallel execution
temp_cluster <- parallel::makeCluster(
  parallel::detectCores() - 1,
  type = "PSOCK"
)
doParallel::registerDoParallel(cl = temp_cluster)

#parallelized loop (each sensor is processed in one separated thread)
moiscrust_interpolation <- foreach::foreach(
  sensor_i = sensors,
  .packages = c("magrittr", "dplyr")
) %dopar% {

  #subset sensors_pairs
  sensors_pair_i <- sensors_pairs %>%
    dplyr::filter(y == sensor_i)

  #fill microsite
  y[, "microsite"] <- sensors_pair_i$y_microsite[1]

  #scanning the rows of x one by one
  for(row_i in 1:nrow(x)){

    #if is not NA, next iteration
    if(!is.na(x[row_i, sensor_i])){next}
```

```r
#getting target row row
x_row_i <- x[row_i, ]

#getting predictor candidates available in x_row_i
predictor_candidates_i <- as.vector(x_row_i)
predictor_candidates_i <- predictor_candidates_i[which(
  !is.na(predictor_candidates_i) &
    names(predictor_candidates_i) != sensor_i
  )]

#selecting the predictor candidate with the best selection_score score
best_predictor_i <- sensors_pair_i %>%
  dplyr::filter(x %in% names(predictor_candidates_i)) %>%
  dplyr::arrange(desc(selection_score)) %>%
  dplyr::slice(1)

#if there is no best candidate available, next iteration
if(nrow(best_predictor_i) == 0){next}

#compute estimates with the model of the best predictor
prediction <- predict(
    object = sensors_pairs_models[[best_predictor_i$model_id]],
    newdata = x_row_i,
    se.fit = TRUE,
    type = "response",
    interval = "confidence"
    )$fit

#replacing negative values with 0
if(prediction[1] < 0){
  prediction[1] <- 0
}
if(prediction[2] < 0){
  prediction[2] <- 0
}
if(prediction[3] < 0){
```

```
    prediction[3] <- 0
   }

   #save prediction
   y[row_i, c(
     "model_estimate",
     "model_ci_lower",
     "model_ci_upper"
     )] <- prediction

   #adding interpolation flag
   y[row_i, "interpolated"] <- TRUE
   y[row_i, "model_predictor"] <- best_predictor_i$x
   y[row_i, "sensors_r_squared"] <- best_predictor_i$sensors_r_squared
   y[row_i, "selection_score"] <- best_predictor_i$selection_score
   y[row_i, "sensors_shared_valid_percent"] <- best_predictor_i$sensors_shared_valid_percent
   y[row_i, "same_microsite"] <- best_predictor_i$same_microsite

  }

  #adding sensor_i name
  y[, "sensor"] <- sensor_i

  return(y)

}

#stop cluster
parallel::stopCluster(temp_cluster)

#removing loop objects
rm(
  x,
  y,
  temp_cluster
)
```

The imputation algorithm produces a list named **moiscrust_interpolation**, with one slot per sensor, each one with one **y** data frame containing the imputation results. Below we transform this object into the data frame **moiscrust_interpolation_long** and join it with **moiscrust_long**, to start preparing the database format.

```
#naming the output
names(moiscrust_interpolation) <- sensors

#to data frame
moiscrust_interpolation_long <- do.call(
  "rbind",
  moiscrust_interpolation
)

#joining with moiscrust_long
moiscrust_long <- dplyr::left_join(
  moiscrust_long,
  moiscrust_interpolation_long,
  by = c("date_time_id", "sensor")
)

#transferring estimates to the soil_moisture column
moiscrust_long$soil_moisture <- ifelse(
  is.na(moiscrust_long$soil_moisture),
  moiscrust_long$model_estimate,
  moiscrust_long$soil_moisture
)

#adding a interpolation_quality flag following the criteria in the paper
moiscrust_long$interpolation_quality <- moiscrust_long$sensors_r_squared

#filling NA with "observation"
moiscrust_long[
  is.na(moiscrust_long$interpolation_quality),
  "interpolation_quality"
  ] <- 1
```

```
#adding NA where there are no values
moiscrust_long[is.na(moiscrust_long$soil_moisture), "interpolation_quality"] <- NA

#computing number of NA cases again
moiscrust_NA <- moiscrust_long %>%
 group_by(sensor) %>%
 summarise(na_count_after = sum(is.na(soil_moisture))) %>%
 mutate(na_count_percent_after = round((na_count_after * 100) / nrow(moiscrust), 1)) %>%
 left_join(
  y = moiscrust_NA,
  by = "sensor"
 ) %>%
 transmute(
  sensor,
  na_count_before = na_count,
  na_count_after,
  na_count_percent_before = na_count_percent,
  na_count_percent_after
 )

#removing moiscrust_interpolation
rm(moiscrust_interpolation)
```

The interpolation has removed all gaps where there was a reference value to interpolate from, as shown in the table below.

Sensor	NA before interpolation	NA after interpolation	NA % before interpolation	NA % after interpolation
bare_soil_1	18442	11338	39.6	24.4
bare_soil_2	27546	16872	59.2	36.3
bare_soil_3	34707	4191	74.6	9.0
biocrust_high_1	22940	12442	49.3	26.7
biocrust_high_2	18813	6558	40.4	14.1
biocrust_high_3	24225	5640	52.1	12.1
biocrust_medium_1	25797	7906	55.5	17.0
biocrust_medium_2	29532	9232	63.5	19.8
biocrust_medium_3	30596	5509	65.8	11.8
retama_1	30111	8967	64.7	19.3
retama_2	31366	7529	67.4	16.2
retama_3	24007	12216	51.6	26.3
stipa_1	20840	10547	44.8	22.7
stipa_2	27698	12885	59.5	27.7
stipa_3	13963	2049	30.0	4.4

4.5 Visualizing the interpolated time series

The **MOISCRUST** database looks as follows after applying the imputation algorithm.

```
ggplot(moiscrust_long) +
  facet_wrap(
    "year",
    scales = "free_x",
    ncol = 2
    ) +
  aes(
    x = year_day,
    y = sensor,
    fill = soil_moisture
    ) +
  geom_tile() +
  coord_cartesian(expand = FALSE) +
  theme_bw() +
  scale_fill_viridis_c(
    direction = -1,
    na.value = "gray50",
    option = "B"
    ) +
  theme(legend.position = "top") +
```

```r
ylab("") +
xlab("Day of the year") +
ggtitle("MOISCRUST database (observed and interpolated records)") +
labs(fill = expression("Volumetric water content (m³ water / m³ soil)")) +
theme(legend.key.width = unit(0.8, "cm"))
```

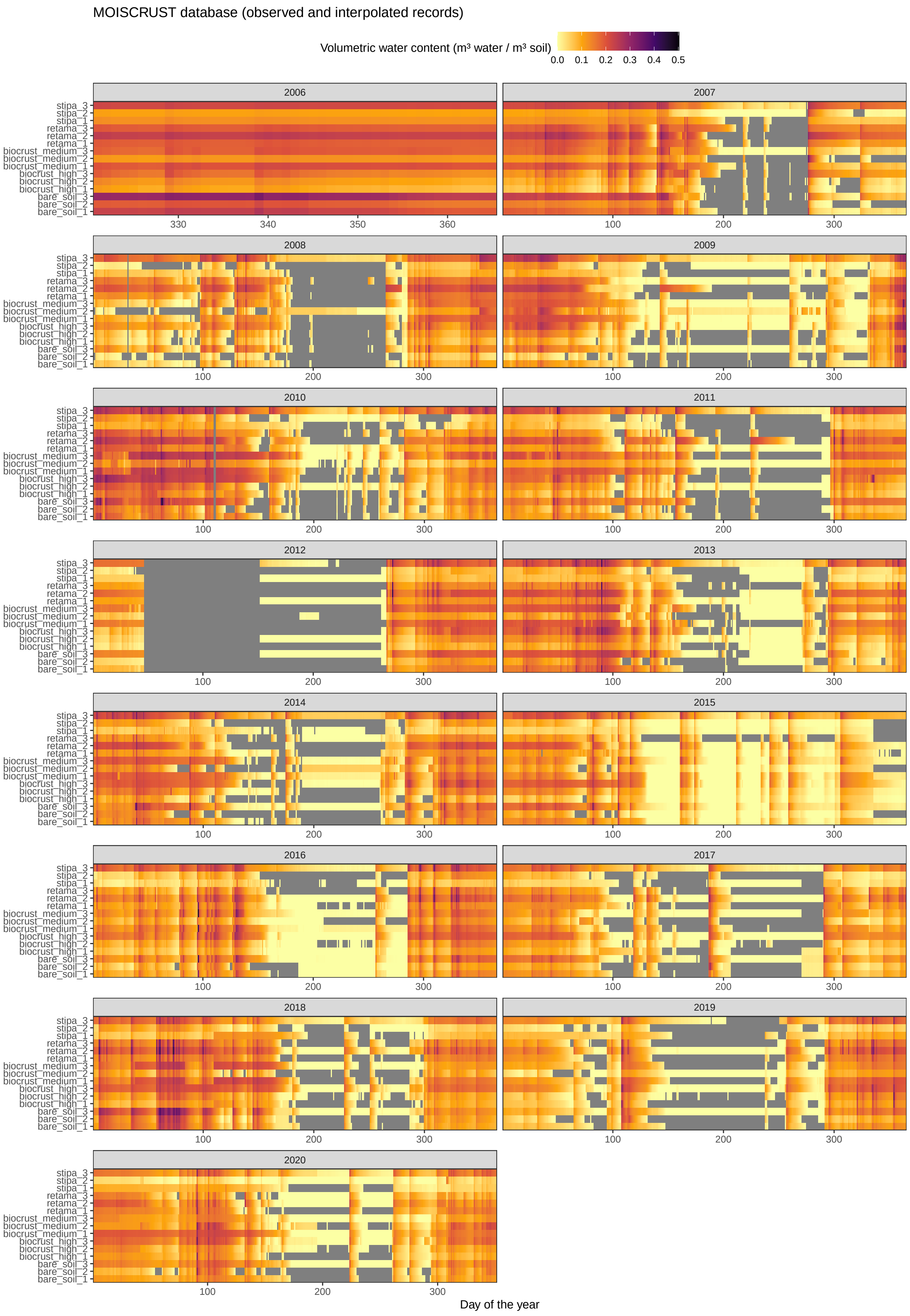
MOISCRUST database (observed and interpolated records)
Volumetric water content (m³ water / m³ soil)
0.0 0.1 0.2 0.3 0.4 0.5
2006
2007
2008
2009
2010
2011
2012
2013
2014
2015
2016
2017
2018
2019
2020
stipa_3
stipa_2
stipa_1
retama_3
retama_2
retama_1
biocrust_medium_3
biocrust_medium_2
biocrust_medium_1
biocrust_high_3
biocrust_high_2
biocrust_high_1
bare_soil_3
bare_soil_2
bare_soil_1
330 340 350 360
100 200 300
Day of the year

The plot above represents both observed and interpolated values. However, the imputation algorithm also generated a new column named *interpolation_quality*, where the observations are marked with the correlation coefficient of the model used to impute each value, as shown in the plot below, where the value 1 (color black) represents actual observations.

```
ggplot(moiscrust_long) +
 facet_wrap(
  "year",
  scales = "free_x",
  ncol = 2
  ) +
 aes(
  x = year_day,
  y = sensor,
  fill = interpolation_quality
  ) +
 geom_tile() +
 coord_cartesian(expand = FALSE) +
 theme_bw() +
 scale_fill_viridis_c(
  direction = -1,
  na.value = "gray50",
  option = "B"
  ) +
 theme(legend.position = "top") +
 ylab("") +
 xlab("Day of the year") +
 ggtitle("MOISCRUST database (data quality)") +
 labs(
  fill = expression("imputation quality")) +
 theme(legend.key.width = unit(1, "cm"))
```

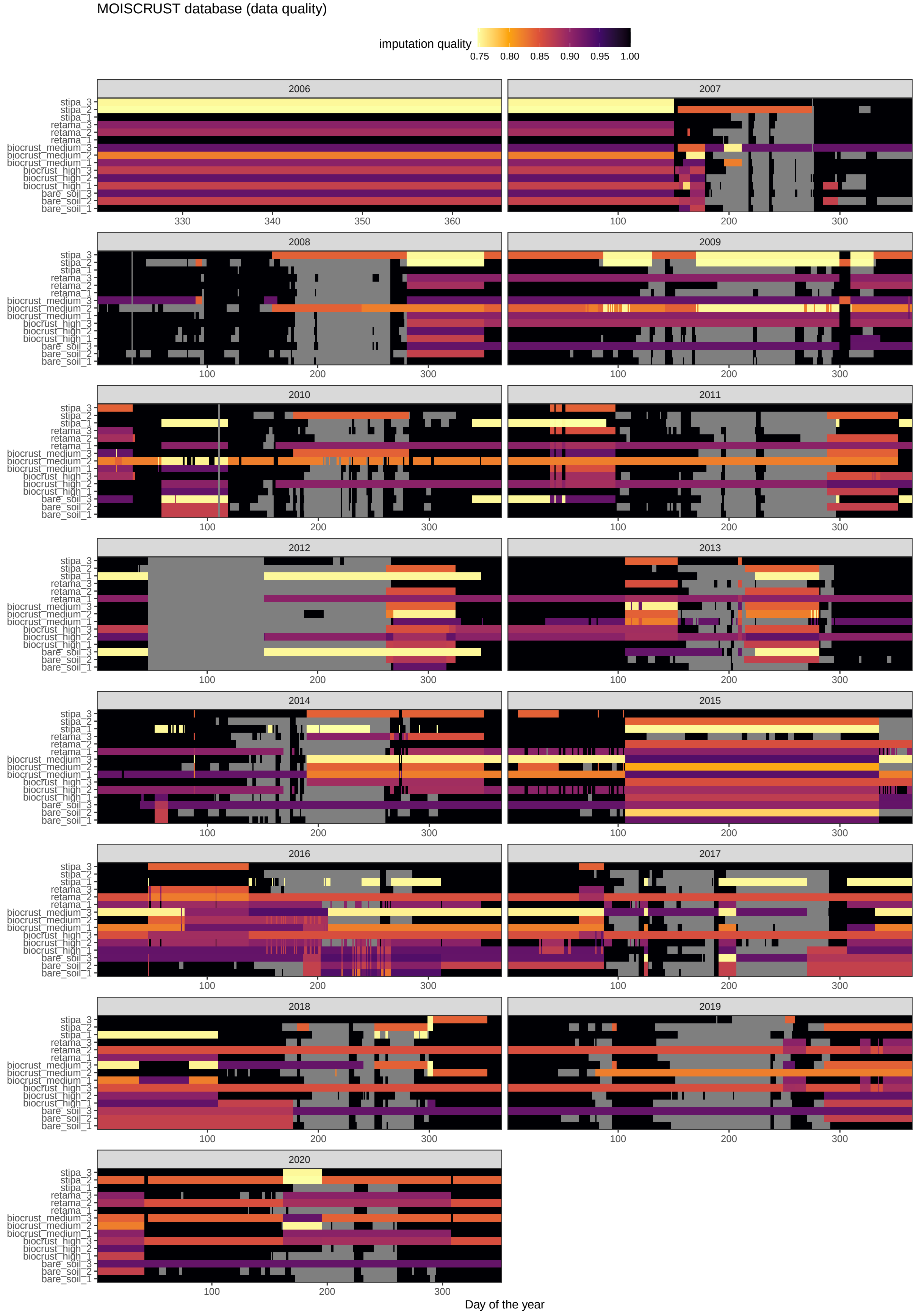
MOISCRUST database (data quality)
imputation quality
0.75
0.80
0.85
0.90
0.95
1.00
2006
2007
2008
2009
2010
2011
2012
2013
2014
2015
2016
2017
2018
2019
2020
stipa_3
stipa_2
stipa_1
retama_3
retama_2
retama_1
biocrust_medium_3
biocrust_medium_2
biocrust_medium_1
biocrust_high_3
biocrust_high_2
biocrust_high_1
bare_soil_3
bare_soil_2
bare_soil_1
330
340
350
360
100
200
300
Day of the year

5 Incorporating weather data at daily resolution

The file data/daily_weather_aranjuez.csv contains daily records of solar radiation (daily sum, in W/m^2), temperature (maximum and minimum, in °C), rainfall (daily sum, in mm.), and humidity (in percentage). To join this file with the moiscrust dataset, here we import it, aggregate moiscrust at daily resolution using only observations, and finally join both datasets by date.

```
#importing the table
weather <- data.table::fread("data/daily_weather_aranjuez.csv") %>%
  as.data.frame()

#formatting date
weather$date <- format(
  as.POSIXct(
    strptime(
      weather$date,
      "%d/%m/%Y",
      tz = ""
    )
  ),
  format = "%Y-%m-%d"
)

#function to compute the mode of a character vector
char_mode <- function(x){
  x.unique <- unique(na.omit(x))
  x.unique[which.max(tabulate(match(x, x.unique)))]
}

#aggregating moiscrust at daily resolution
moiscrust_daily <- moiscrust_long %>%
  dplyr::group_by(sensor, year, year_day) %>%
  dplyr::summarise(
    date = date[1],
    month = month[1],
```

```
    month_day = month_day[1],
    week = week[1],
    week_day = week_day[1],
    microsite = microsite[1],
    soil_moisture_min = suppressWarnings(min(soil_moisture, na.rm = TRUE)),
    soil_moisture_mean = mean(soil_moisture, na.rm = TRUE),
    soil_moisture_max = suppressWarnings(max(soil_moisture, na.rm = TRUE)),
    interpolated = ifelse(sum(interpolated) > 0, TRUE, FALSE),
  ) %>%
  dplyr::filter(interpolated == FALSE) %>%
  dplyr::select(-interpolated) %>%
  as.data.frame()

#NaN to NA
is.nan.data.frame <- function(x){
  do.call(cbind, lapply(x, is.nan))
}
moiscrust_daily[is.nan(moiscrust_daily)] <- NA

#joining by date
moiscrust_weather <- dplyr::left_join(
  moiscrust_daily,
  weather,
  by = "date"
) %>%
  dplyr::select(
    -contains("model_"),
    -year.y,
    -month.y
    ) %>%
  dplyr::rename(
    year = year.x,
    month = month.x,
    temperature_max = temperature_maximum,
    temperature_min = temperature_minimum
  ) %>%
```

```
dplyr::transmute(
  date,
  year,
  year_day,
  season,
  month,
  month_day,
  week,
  week_day,
  sensor,
  microsite,
  soil_moisture_min,
  soil_moisture_mean = round(soil_moisture_mean, 3),
  soil_moisture_max,
  solar_radiation_sum,
  temperature_max,
  temperature_min,
  rainfall_sum,
  humidity_average
) %>%
na.omit()
```

The resulting table, `moiscrust_weather`, has daily observations of soil humidity (not imputed data) coupled with daily weather data.

6 Preparing database formats

6.1 Format description

6.1.1 moiscrust

The dataset **moiscrust_long** is the MOISCRUST database in long format Below we rename it to **moiscrust**, reorder its columns, and describe its structure.

```r
moiscrust <- moiscrust_long[ , c(
  "date_time",
  "date_time_id",
  "date",
  "time",
  "year",
  "year_day",
  "month",
  "week",
  "week_day",
  "sensor",
  "microsite",
  "soil_moisture",
  "interpolated",
  "interpolation_quality",
  "model_ci_lower",
  "model_ci_upper",
  "model_predictor",
  "same_microsite",
  "sensors_shared_valid_percent",
  "selection_score"
)]
```

The columns of the `moiscrust` data frame are:

- *date_time*: date and time in POSIX format.
- *date_time_id*: integer, unique ID for each value of *date_time*.
- *date*: date in format year-month-day.
- *time*: time in format hour-minute.
- *year*: integer, year.
- *year_day*: integer, day of the year.
- *month*: integer, month number.
- *week*: integer, week of the year.
- *week_day*: integer, day of the week.
- *sensor*: character, sensor name.
- *microsite*: character, name of the microsite.

- *soil_moisture*: numeric, soil moisture value in $m^3 water/m^3 soil$.
- *interpolated*: boolean, *TRUE* for interpolated records and *FALSE* for observations.
- *interpolation_quality*: numeric, r-squared of the model used to interpolate the soil moisture value.
- *model_ci_lower*: numeric, lower bound of the confidence interval of the estimate.
- *model_ci_upper*: numeric, upper bound of the confidence interval of the estimate.
- *model_predictor*: character, name of the sensor used as predictor in the linear model.
- *same_microsite*: boolean, *TRUE* if the sensor and its predictor are in the same microsite group ("stipa", "retama", "biocrust_low", "biocrust_medium", "biocrust_high").
- *sensors_shared_valid_percent*: numeric, percentage of shared valid cases between *sensor* and *model_predictor*, taking the total number of values in *date_time_id* as reference.
- *selection_score*: numeric, value used to select the *model_predictor*, based on the sum of *same_microsite* (100 if *TRUE* and 0 if *FALSE*), *sensors_r_squared* multiplied by 100, and *sensors_shared_valid_percent*.

6.1.2 moiscrust_weather

The moiscrust_weather data frame, at daily resolution, has the following columns:

- *date*: date in format year-month-day.
- *year*: integer, year.
- *year_day*: integer, day of the year.
- *season*: character, name of the season.
- *month*: integer, month number.
- *month_day*: integer, day of the month.
- *week*: integer, week of the year.
- *week_day*: integer, day of the week.
- *sensor*: character, sensor name.
- *microsite*: character, name of the microsite.
- *soil_moisture_min*: numeric, minimum soil moisture value for the given day.
- *soil_moisture_mean*: numeric, mean soil moisture for the given day.
- *soil_moisture_max*: numeric, maximum soil moisture registered for the given day.
- *solar_radiation_sum*: numeric, sum of daily solar radiation, in W/m^2.

- *temperature_max*: numeric, maximum daily temperature, in C.
- *temperature_min*: numeric, minimum daily temperature, in C.
- *rainfall_sum*: numeric, total daily rainfall, in mm.
- *humidity_average*: numeric, daily average humidity, in .

6.2 Saving the data base in different formats

To expand its usability as much as possible, we provide the data in four different formats: .RData, .csv, .xlsx, and .db (SQLite). The output files are written first to the database folder, that is later compressed and named database.zip.

```
#if the zip file does not exists, creates database directory and populates it
if(!file.exists("database.zip")){

 dir.create("database")

 #save as RData
 save(
  moiscrust,
  moiscrust_weather,
  file = "database/moiscrust.RData"
 )

 #save as csv
 readr::write_excel_csv(
  x = moiscrust,
  path = "database/moiscrust.csv"
 )
 readr::write_excel_csv(
  x = moiscrust_weather,
  path = "database/moiscrust_weather.csv"
 )

 #save as excel file
 writexl::write_xlsx(
  x = list(
```

```
    moiscrust = moiscrust,
    moiscrust_weather = moiscrust_weather
    ),
  path = "database/moiscrust.xlsx"
 )

 #save as SQLite
 db.driver <- DBI::dbDriver("SQLite")
 db.connection <- DBI::dbConnect(
  db.driver,
  dbname = "database/moiscrust.db"
  )
 DBI::dbWriteTable(
  db.connection,
  "moiscrust",
  moiscrust,
  overwrite = TRUE
  )
  DBI::dbWriteTable(
  db.connection,
  "moiscrust_weather",
  moiscrust_weather,
  overwrite = TRUE
  )
 DBI::dbDisconnect(db.connection)

 #compressing the file
 zip::zipr(
  zipfile = "database.zip",
  files = "database"
 )

}
```
